# The relationship between sclerostin and carotid artery atherosclerosis in patients with stage 3–5 chronic kidney disease

**DOI:** 10.1007/s11255-020-02495-x

**Published:** 2020-05-26

**Authors:** Ban Zhao, Aiqun Chen, Haitao Wang, Ju Cui, Ying Sun, Lengnan Xu, Yonghui Mao

**Affiliations:** 1 The Department of Nephrology, Beijing Hospital, National Center of Gerontology, Institute of Geriatric Medicine, Chinese Academy of Medical Sciences, NO. 1 DaHua Road, Dong Dan, Beijing, 100730 People’s Republic of China; 2 The Key Laboratory of Geriatrics, Beijing Institute of Geriatrics, Beijing Hospital, National Center of Gerontology, National Health Commission, Institute of Geriatric Medicine, Chinese Academy of Medical Sciences, Beijing, People’s Republic of China

**Keywords:** Atherosclerosis, Chronic kidney disease, Renal function, Sclerostin, Wnt pathway

## Abstract

**Purpose:**

Sclerostin is an antagonist of the Wnt/β-catenin pathway. We previously reported that sclerostin is closely related to carotid artery atherosclerosis and long-term outcome in hemodialysis patients. The present study investigated the association between sclerostin, renal function, and carotid artery atherosclerosis in non-dialysis patients with stage 3–5 chronic kidney disease (CKD 3–5ND).

**Methods:**

A total of 140 patients with CKD 3–5ND were enrolled in this cross-sectional study. The Chronic Kidney Disease Epidemiology Collaboration equation was used to calculate estimated glomerular filtration rate (eGFR). Atherosclerotic plaques in the carotid artery were detected by B-mode Doppler ultrasound. Blood samples were collected to assess serum sclerostin levels. Unconditional logistic regression analysis was used to identify risk factors for carotid atherosclerotic plaques.

**Results:**

The median eGFR was 24.9 ml/min/1.73 m^2^ (interquartile range [IQR] 10.0–40.3 ml/min/1.73 m^2^) and median serum sclerostin level was 46.76 pmol/l (IQR 30.18–67.56 pmol/l). Carotid atherosclerotic plaques were detected in 104 subjects (74.3%). There was a negative association between sclerostin level and eGFR (*r* =  − 0.214, *p* = 0.011). Unconditional logistic regression analysis revealed that sclerostin level was an independent risk factor for the occurrence of carotid plaques, with an odds ratio (95% confidence interval) of 1.026 (1.003, 1.051).

**Conclusion:**

Serum sclerostin increases with declining renal function in patients with CKD 3–5ND. Sclerostin is an independent risk factor for carotid atherosclerosis.

**Electronic supplementary material:**

The online version of this article (10.1007/s11255-020-02495-x) contains supplementary material, which is available to authorized users.

## Introduction

Cardiovascular disease is more prevalent in patients with chronic kidney disease (CKD) than in the general population [1, 2]. The Wnt/β-catenin signaling pathway plays an important role in the pathophysiology of atherosclerosis [3–5]. The Wnt-β-catenin signaling pathway is an important player in bone remodeling, and is involved in osteoblast proliferation, differentiation and bone formation [6, 7]. Dysregulation of the Wnt-β-catenin pathway also plays a crucial role in chronic kidney disease–mineral bone disorder (CKD-MBD) [6]. Sclerostin encoded by the *SOST* gene is an antagonist of the Wnt/β-catenin pathway that is mainly secreted by osteoblasts and inhibits bone formation [6]. Inactivating mutations in the *SOST* gene in mice were shown to increase bone mass [8], whereas activating mutations resulted in bone loss [9]. Monoclonal antibodies against sclerostin have been used to treat osteoporosis in postmenopausal women, resulting in a dose-dependent increase in bone mineral density [10]. Serum levels of sclerostin are higher in CKD patients than in the general population and begin increasing during stage 3 [11]. However, it remains unclear how increased sclerostin relates to abnormalities in bone turnover in CKD patients.

Sclerostin has been detected on the surface of mineralized osteoblast-like cells in vitro and in the calcified aortic valve tissue of patients undergoing hemodialysis (HD) [12, 13], as well as in carotid atherosclerotic plaques by immunohistochemistry [14]. Clinical studies have reported a correlation between serum sclerostin levels and atherosclerosis in obese and diabetic patients [15, 16]. Based on this evidence, we hypothesized that sclerostin plays an important role in the pathophysiology of atherosclerosis. Few studies have examined the correlation between serum sclerostin level and atherosclerosis in non-dialysis patients with CKD (CKD-ND) [17]. Here we investigated the relationship between sclerostin and atherosclerosis in non-dialysis patients with stage 3–5 CKD (CKD 3–5ND).

## Methods

### Study population

A total of 140 patients aged ≥ 18 years with CKD 3–5ND were enrolled in the study between February 2015 and October 2016. Patients on systemic immunosuppressive medication or with active cancer or liver disease, malignant hematologic disorders, acute renal failure, fractures, and/or acute or chronic infections were excluded.

The detailed medical history including age, sex, height, weight, and cause of CKD (chronic glomerulonephritis, hypertensive renal disease, diabetic nephropathy, chronic interstitial nephritis, polycystic kidney disease, autoimmune disease, or other disease) were recorded. We also obtained information related to medical history, smoking (patients who had stopped smoking for > 5 years were classified as non-smokers), diabetes mellitus (DM), and hypertension (including primary and renal hypertension). The study protocol was approved by the ethics committee of Beijing Hospital (no. 2014BJYYEC-058-01), and written informed consent was obtained from all patients.

### Assessment of kidney function

Estimated glomerular filtration rate (eGFR) was calculated using the Chronic Kidney Disease Epidemiology Collaboration equation, as shown below:

$${\text{eGFR}} = {175 } \times \, \left[ {\text{serum creatinine}} \right]^{{ - {1}.{154}}} \times {\text{ age}}^{{ - 0.{2}0{3}}} \left( { \times \, 0.{742},{\text{ if female}}} \right).$$

The eGFR data were used to group patients according to CKD stage as follows: stage 3 (eGFR: 30–60 ml/min/1.73 m^2^), stage 4 (eGFR: 15–30 ml/min/1.73 m^2^), and stage 5 (eGFR: < 15 ml/min/1.73 m^2^).

### Biochemical analyses

Venous blood samples were collected in a fasting state, and serum creatinine levels were measured with the enzymatic isotope dilution mass spectrometry traceable standardized method (Cobas C501 Biochemical Analyzer; Roche Diagnostics, Mannheim, Germany). Serum levels of uric acid, total serum calcium, phosphate, albumin, total cholesterol, low-density lipoprotein cholesterol (LDL-C), high-density lipoprotein cholesterol (HDL-C), alkaline phosphatase (ALP), high-sensitivity C-reactive protein (hs-CRP), intact parathyroid hormone (iPTH), and hemoglobin were measured according to standard methods at the hospital’s central laboratory. Serum samples were then stored at − 80 °C, an enzyme-linked immunosorbent assay kit (Biomedica, Wien, Austria) was used to determine serum levels of sclerostin at October 2016. A total of 18 patients with normal renal function (11 male, seven females; mean age, 59.9 years) were recruited for the control group; the median sclerostin level in these subjects was 36.31 (26.29, 43.60) pmol/l. Baseline serum samples of the 140 patients were stored at − 80 °C, the levels of 25 hydroxyvitamin D were tested in March 2020 (Cobas e601 automatic electrochemiluminescence analyzer, Roche Diagnostics, Mannheim, Germany).

### B mode and Doppler ultrasound imaging of common carotid artery

Baseline B mode and Doppler ultrasound images of the common carotid artery were obtained. An atherosclerotic plaque was defined as a focal structure encroaching into the arterial lumen by at least 0.5 mm or 50% of the surrounding intima-media thickness value, or with a thickness > 1.5 mm, as measured from the media-adventitia interface to the intima-lumen interface [18]. The sonographer searched for plaques by scanning the common carotid artery and its bifurcation, proximal internal carotid artery, and external carotid artery segment in both longitudinal and cross-sectional planes [19]. Bilateral carotid arteries were analyzed by color Doppler ultrasound at a frequency of 5–10 MHz (Model IU-22; Philips, Amsterdam, The Netherlands) by an experienced sonographer, with each measurement repeated twice.

### Statistical analyses

Statistical analyses were performed with SPSS v20.0 software (IBM, Armonk, NY, USA). The normality of raw data was assessed with the Kolmogorov–Smirnov test. Normally and non-normally distributed continuous variables are expressed as mean ± standard deviation and median with 25th and 75th percentiles, respectively. Differences between groups were evaluated with the Student’s *t* test or the Mann–Whitney *U* test depending on whether the data were normally distributed. Categorical data are reported as percentages and were assessed with the chi-squared test. Spearman’s method was used to analyze the correlation between sclerostin level and other parameters. Risk factors for carotid atherosclerotic plaques were evaluated by unconditional logistic regression. For all analyses, *p* < 0.05 was considered as statistically significant.

## Results

### Baseline patient characteristics

The demographic and clinical characteristics of the study population are shown in Table 1. A total of 140 subjects (age range 25–81 years) were enrolled, including 60 patients with DM (44.3%). Among these patients, the causes of CKD were chronic glomerulonephritis (*n* = 41, 29.3%), hypertensive renal disease (*n* = 39, 27.9%), diabetic nephropathy (*n* = 33, 23.6%), chronic interstitial nephritis (*n* = 15, 10.7%), polycystic kidney disease (*n* = 3, 2.1%), autoimmune diseases (*n* = 2, 1.4%), and other diseases (*n* = 7, 5.0%). There were 58 patients in CKD stage 3 (41.4%), 36 in stage 4 (25.7%), and 46 in stage 5 (32.9%).

**Table 1:** The relationships between serum sclerostin level and renal function and bone and mineral metabolism

Variable	*r*	*p* value
eGFR, ml/min/1.73 m^2^	− 0.214	0.011
Calcium, mmol/L	− 0.225	0.007
Phosphate, mmol/L	0.185	0.028
ALP, U/L	− 0.150	0.078
Intact parathyroid hormone, pg/mL	0.098	0.250
25(OH) vitamin D, ng/ml	− 0.075	0.378

The median eGFR was 24.9 ml/min/1.73 m^2^, and median serum sclerostin concentration was 46.76 pmol/l. Carotid atherosclerotic plaques were detected in 104 subjects (74.3%). Males had significantly higher serum sclerostin levels than females (57.26 vs 43.05 pmol/l; median, *p* < 0.001). Higher sclerostin levels were observed in patients with a history of smoking (59.08 vs 45.93 pmol/l; median, *p* = 0.063) and hypertension (47.75 vs 34.41 pmol/l; median, *p* = 0.056) compared to non-smokers and patients with no history of hypertension. Sclerostin levels were comparable between patients with and those without DM (47.28 vs 45.75 pmol/l; median, *p* = 0.273).

### Relationships between serum sclerostin level and renal function and bone and mineral metabolism

**Fig. 1:**
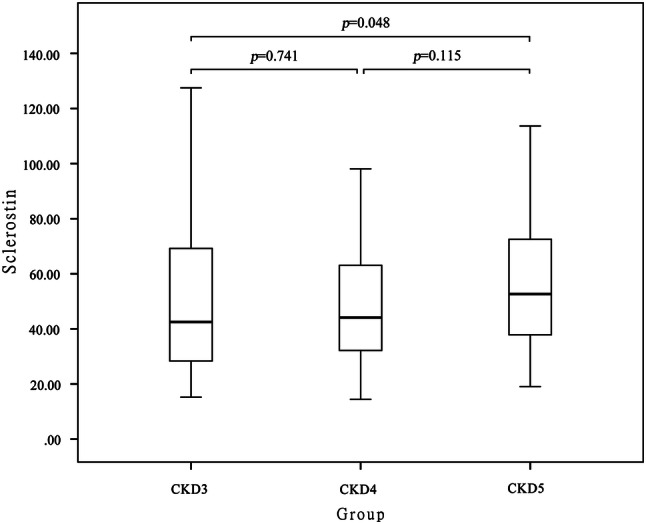
Comparison of serum sclerostin between patients in CKD 3, 4 and 5 stages

Serum sclerostin levels gradually increased with the deterioration of renal function. A Spearman correlation analysis showed that serum sclerostin was negatively correlated with eGFR (*r* =  − 0.214, *p* = 0.011), and a higher level was observed in patients with stage 5 as compared to stage 3 CKD (42.53 vs 52.64 ng/ml, median, *p* = 0.048), although the levels were comparable between stages 3 and 4 (42.53 vs 44.11 ng/ml, *p* = 0.741) and between stages 4 and 5 (44.11 vs 52.64 ng/ml, *p* = 0.115) (Fig. 1). Spearman correlation analysis showed that serum sclerostin was negatively correlated with calcium (*r* =  − 0.225, *p* = 0.007) but positively correlated with phosphorus (*r* = 0.185, *p* = 0.028). There was no correlation between serum sclerostin and iPTH, hs-CRP, 25(OH) vitamin D, and alkaline phosphatase (Table 1). Sclerostin level was non-normally distributed and was log transformed, and multiple stepwise regression analysis was carried out with sex, age, body mass index (BMI), eGFR, iPTH, 25(OH) vitamin D, serum calcium, phosphorus, DM, and atherosclerosis as covariates. The results showed that sex (*p* = 0.001), phosphorus (*p* < 0.001), and atherosclerosis (*p* = 0.001) were independently associated with serum sclerostin level.

### Comparison between high and low sclerostin groups

The subjects (*n* = 140) were divided into high and low sclerostin groups according to median levels (46.76 pmol/l) as in previous studies. The high sclerostin group had more male patients (*p* = 0.002) and higher levels of serum phosphate (*p* = 0.042), and lower eGFR (*p* = 0.020), serum total calcium (*p* = 0.007), hemoglobin (*p* = 0.008), and ALP (*p* = 0.034) (Table 2).

**Table 2:** Demographic and clinical characteristics of the patients involved in this study along with comparisons between patients in the high and low sclerostin groups

Variable	All patients *n* = 140	Sclerostin < 46.76 pmol/L *n* = 70	Sclerostin > 46.76 pmol/L *n* = 70	*p* value
Age (years)	64 (51, 73)	64 (49, 74)	64 (52, 73)	0.995
Male, *n* (%)	72 (51.4)	27 (38.6)	45 (64.3)	0.002
Diabetes, *n* (%)	60 (44.3)	29 (41.4)	31 (44.3)	0.733
Hypertension, *n* (%)	120 (85.7)	57 (81.4)	63 (90.0)	0.147
Atherosclerotic plaque, *n* (%)	104 (74.3)	50 (71.4)	54 (77.1)	0.439
Smoker, *n* (%)	38 (27.1)	16 (22.9)	22 (31.4)	0.254
BMI (kg/m^2^)	24.82 ± 3.91	25.00 ± 3.46	24.65 ± 4.33	0.601
Systolic BP (mmHg)	130 (130, 150)	133 (130, 150)	130 (130, 150)	0.594
Diastolic BP (mmHg)	80 (70, 86)	80 (70, 90)	80 (70, 80)	0.266
Pulse pressure (mmHg)	60 (50, 70)	60 (50, 65)	60 (50, 70)	0.431
eGFR (mL/min/1.73 m^2^)	24.9 (10.0, 40.3)	26.8 (14.3, 44.3)	22.0 (8.0, 36.8)	0.020
Hemoglobin (g/L)	110 ± 25	115 ± 23	104 ± 26	0.008
Albumin (g/L)	40 (37, 43)	41 (38, 43)	40 (36, 42)	0.050
Phosphate (mmol/L)	1.37 (1.17, 1.68)	1.32 (1.18, 1.52)	1.45 (1.16, 1.82)	0.042
iPTH (pg/mL)	85 (47, 189)	79 (45, 179)	103 (50, 207)	0.161
25 (OH) vitamin D (ng/ml)	8.3 (4.6, 12.0)	9.6 (5.5, 13.2)	7.2 (4.2, 11.7)	0.141
Alkaline phosphatase (U/L)	75 (59, 92)	81 (59, 97)	67 (59, 83)	0.034
Calcium (mmol/L)	2.23 (2.10, 2.34)	2.28 (2.16, 2.34)	2.18 (2.00, 2.32)	0.007
Uric acid (umol/L)	442 ± 126	424 ± 117	460 ± 133	0.086
Cholesterol (mmol/L)	4.31 ± 0.95	4.37 ± 0.98	4.25 ± 0.93	0.429
LDL-C (mmol/L)	2.54 ± 0.75	2.56 ± 0.71	2.52 ± 0.78	0.738
HDL-C (mmol/L)	1.08 (0.91, 1.28)	1.13 (0.91, 1.35)	1.07 (0.91, 1.25)	0.528
hs-CRP (mg/dl)	1.84 (0.85, 4.67)	1.84 (0.61, 4.13)	1.82 (0.86, 6.94)	0.250
Anti-hypertensive drug, *n* (%)	136 (97.1)	68 (97.1)	68 (97.1)	1.000
Statin, *n* (%)	86 (61.4)	48 (68.6)	38 (54.3)	0.083
Calcium-based phosphate binders, *n* (%)	28 (20.0)	15 (21.4)	13 (18.6)	0.673
Calcitriol, *n* (%)	43 (30.7)	27 (38.6)	16 (22.8)	0.044

Normally distributed variables are shown as mean ± standard deviation; non-normally distributed variables are shown as medians (with 25 and 75% interquartile ranges in parentheses)

*BMI* body mass index, *eGFR* estimated glomerular filtration rate, *iPTH* intact parathyroid hormone, *LDL-C* low-density lipoprotein cholesterol, *HDL-C* high-density lipoprotein cholesterol, *hs-CRP* high-sensitivity C-reactive protein

### Comparison between patients with and without atherosclerotic plaques

**Fig. 2:**
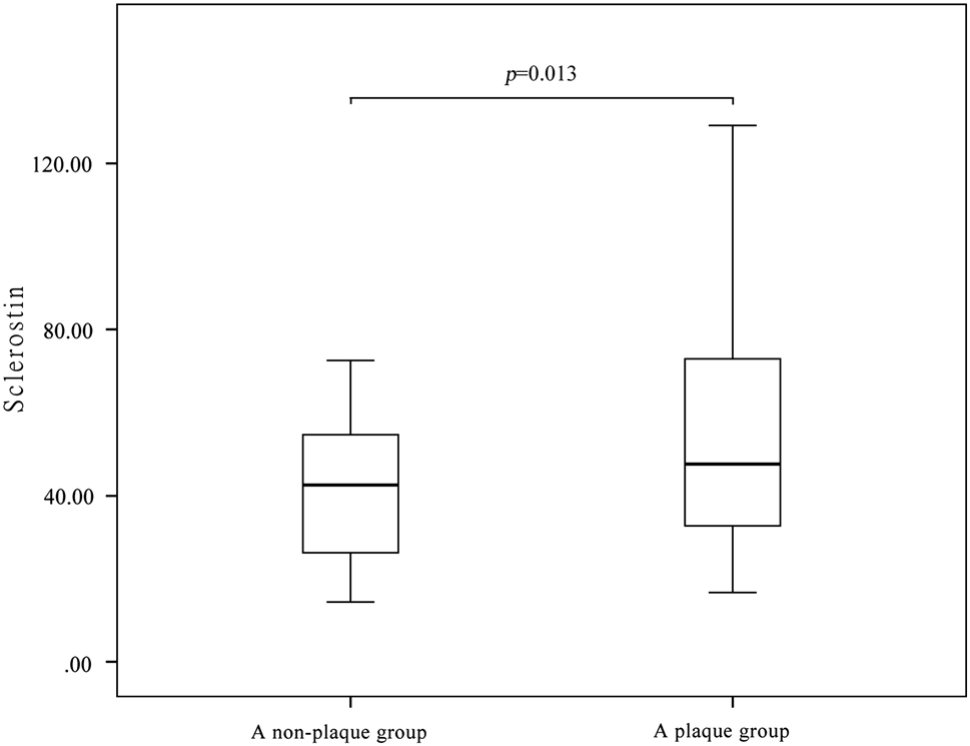
Comparison of serum sclerostin between patients in the plaque group and the non-plaque group

Subjects were divided into plaque (*n* = 104) and non-plaque (*n* = 36) groups according to the presence of carotid atherosclerotic plaques. The plaque group had higher serum levels of sclerostin (*p* = 0.013) (Fig. 2), were older (*p* < 0.001), and had a higher pulse pressure (*p* = 0.036), and higher prevalence of hypertension (*p* = 0.007) and DM (*p* < 0.001) compared to the non-plaque group. Meanwhile, the proportion of patients taking statins was higher in the plaque group (70.2 vs. 36.1%, *p* < 0.001) (Table 3).

**Table 3:** Comparisons between patients with and without atherosclerotic plaques

Variable	Patients with plaques *n* = 104	Patients without plaques *n* = 36	*p* value
Age (years)	67.0 (61.0, 74.0)	44.5 (35.3, 55.3)	< 0.001
Male, *n* (%)	58 (55.8)	14 (38.9)	0.081
Diabetes, *n* (%)	54 (51.9)	6 (16.7)	< 0.001
Hypertension, *n* (%)	94 (90.4)	26 (72.2)	0.007
Smoker, *n* (%)	32 (30.8)	6 (16.7)	0.101
BMI (kg/m^2^)	25.35 ± 3.72	23.31 ± 4.11	0.007
Systolic BP (mmHg)	130 (130, 150)	137 (130, 150)	0.896
Pulse pressure (mmHg)	60 (50, 70)	50 (45, 60)	0.036
eGFR (mL/min/1.73 m^2^)	11.8 (27.0, 40.7)	5.6 (18.3, 38.0)	0.069
Hemoglobin (g/L)	113 ± 23	101 ± 27	0.013
Albumin (g/L)	41 (38, 43)	40 (37, 43)	0.517
Phosphate (mmol/L)	1.37 (1.11, 1.60)	1.39 (1.19, 1.92)	0.107
iPTH (pg/mL)	77.9 (46.0, 172.3)	114.5 (58.0, 243.8)	0.037
25 (OH) vitamin D (ng/ml)	9.3 (4.6, 13.6)	7.2 (4.8, 10.4)	0.134
Alkaline phosphatase (U/L)	75 (59, 93)	74 (57, 91)	0.543
Calcium (mmol/L)	2.26 (2.13, 2.34)	2.17 (1.97, 2.33)	0.144
Uric acid (umol/L)	418.8 ± 107.3	509.7 ± 150.8	0.002
Cholesterol (mmol/L)	4.26 ± 0.94	4.46 ± 0.99	0.265
LDL-C (mmol/L)	2.51 ± 0.75	2.64 ± 0.75	0.379
HDL-C (mmol/L)	1.07 (0.92, 1.28)	1.13 (0.89, 1.35)	0.894
hs-CRP (mg/dl)	1.80 (0.83, 5.07)	2.02 (1.20, 3.73)	0.635
Sclerostin (pmol/L)	47.66 (32.60, 72.91)	42.62 (26.20- 55.50)	0.013
Anti-hypertensive drug, *n* (%)	100 (96.2)	36 (100.0)	0.233
Statin, *n* (%)	73 (70.2)	13 (36.1)	< 0.001
Calcium-based phosphate binders, *n* (%)	20 (19.2)	8 (22.2)	0.699
Calcitriol, *n* (%)	33 (31.7)	10 (27.8)	0.658

Normally distributed variables are shown as mean ± standard deviation; non-normally distributed variables are shown as medians (with 25 and 75% interquartile ranges in parentheses)

*BMI* body mass index, *eGFR* estimated glomerular filtration rate, *iPTH* intact parathyroid hormone, *LDL-C* low-density lipoprotein cholesterol, *HDL-C* high-density lipoprotein cholesterol, *hs-CRP* high-sensitivity C-reactive protein

### Factors related to carotid atherosclerotic plaques

Unconditional logistic regression analysis was used to identify factors related to carotid atherosclerotic plaques. Age, BMI, DM, hypertension, pulse pressure, eGFR, and sclerostin (*p* < 0.05) were independent variables and the presence of carotid atherosclerotic plaques was the dependent variable. The results showed that age, BMI, DM, and sclerostin were independent factors that were significantly related to the presence of carotid plaques, with odds ratios (95% confidence interval) of 1.136 (1.082, 1.192), 1.170 (1.000, 1.369), 3.372 (1.020, 11.142), and 1.026 (1.003,1.051), respectively (Table 4).

**Table 4:** Factors related to carotid atherosclerotic plaques

Variable	Coefficient (*r*) or *β*	*p*	OR (OR 95% CI)
Age (per year)	0.127	< 0.001	1.136 (1.082, 1.192)
BMI (kg/m^2^)	0.157	0.049	1.170 (1.000, 1.369)
Diabetes (Y versus N)	1.125	0.046	3.372 (1.020, 11.142)
Sclerostin (1 pmol/L)	0.026	0.029	1.026 (1.003, 1.051)

*BMI* body mass index, *OR* odds ratios, *CI* confidence interval

## Discussion

The results of this study indicate that serum sclerostin level is inversely related to renal function, and reveal an association between serum sclerostin level and carotid plaques in patients with CKD 3–5ND. Although sclerostin level has been shown to increase with declining renal function [11, 20], the mechanistic basis for elevated serum sclerostin levels in CKD patients has not been elucidated. It was reported that urinary sclerostin excretion increases with decreasing eGFR, while higher sclerostin levels in CKD patients were unrelated to a decline in renal function [21]. CKD patients have a higher percentage of sclerostin-positive osteocytes than healthy subjects (38% and 26% in stage 2–3 and stage 4 CKD, respectively, vs 5.3% in the control group), which may be due in part to increased sclerostin production by osteocytes [22]. An immunohistochemical analysis showed that serum sclerostin level in calcified aortic valves of HD patients was closely associated with vessel calcification, suggesting that sclerostin maybe originates from an extra-skeletal source [13].

Atherosclerotic plaque formation is a complex process that involves vascular calcification, inflammation, endothelial dysfunction, and the proliferation and migration of vascular smooth muscle cells (VSMCs) [3]. Wnt-β-catenin signaling plays an important role in the pathophysiology of atherosclerosis and regulates endothelial inflammation [23], mesenchymal stem cell differentiation [24, 25], and the proliferation, migration and survival of VSMCs [4]. Wnt protein is also known to promote the adhesion of monocytes to endothelial cells [26]. Missense mutations in the Wnt receptor LDL receptor-related protein 6 have been linked to an increased incidence of early-onset coronary artery disease, hypertension, high-serum LDL, and DM [27]. Sclerotin is a soluble antagonist of the Wnt-β-catenin pathway that disrupts signal transduction by blocking the binding of Wnt ligand to the transmembrane receptor complex [6]. Sclerostin levels were found to be elevated in the media relative to the intima of blood vessels and in vascular smooth muscle cells compared to infiltrating macrophages [14]. Sclerostin may maintain aortic homeostasis by inhibiting inflammation and degradation of the extracellular matrix [28]; moreover, both the transgenic introduction of human sclerostin, and the administration of recombinant mouse sclerostin into apolipoprotein E-deficient mice inhibited angiotensin II-induced aneurysm formation and atherosclerosis [28]. In the present study, sclerostin was independently associated with atherosclerotic plaques, which is consistent with observations in HD patients [29, 30]. Thus, sclerostin may play an important role in the process of atherosclerosis. We speculate that the elevated sclerostin may partly originate from atherosclerotic plaques in CKD patients, which suppresses atherosclerosis in a negative feedback mechanism by inhibiting Wnt signaling, thereby protecting against plaque development. Further studies are needed to determine the precise contributions of sclerostin and the Wnt-β-catenin axis to atherosclerosis.

iPTH inhibits sclerostin expression in osteocytes. Evidence from patients with DM and primary hyperparathyroidism has revealed a negative relationship between serum iPTH and sclerostin levels [31]. Clinical studies have yielded inconclusive findings regarding the relationship between circulating sclerostin levels and iPTH in CKD patients. In dialysis patients, sclerostin was negatively correlated with iPTH [32, 33], which may be related to the suppression of sclerostin production by osteocytes, or the decrease of bone mass in secondary hyperparathyroidism [32]. However, we did not observe a correlation between iPTH and sclerostin in CKD patients prior to dialysis, in agreement with previous reports [11, 20]. It is possible that any suppressive effect by iPTH was masked by its low levels in early-stage CKD and elderly CKD patients. In cases of advanced CKD, sclerostin and iPTH levels are elevated and osteocytes may become resistant to the suppressive effects of the latter. Moreover, other factors such as phosphorus and fibroblast growth factor 23 directly or indirectly regulate sclerostin in CKD [34, 35]. It is also possible that sclerostin is upregulated by as-yet unknown factors under uremic conditions.

It was shown that sclerostin has a direct influence on mineral metabolism, compared to wild-type mice (WT mice), urinary excretion of calcium and the fractional excretion of calcium were decreased in sclerostin knockout mice (sost KO mice) [36]. Furthermore, in the sost KO mice, the serum concentrations of intact FGF-23 were significantly decreased, and the concentrations of the 1-α, 25(OH)_2_D were increased [36]. In a mouse model of CKD, 1-α, 25(OH)_2_D production in osteocytes was shown to stimulate sclerostin and inhibit BMP2 production [37]. Barbara et al. has found plasma sclerostin concentration positively correlated with serum 25-OH-Vitamin D in hemodialysis patients [32]. However, we have found no correlation between serum sclerostin and 25-OH-Vitamin D in the present study. In a model of CKD with adynamic osteopathy, increased bone expression and blood level of sclerostin were closely related to dietary phosphorus intake [38]. In a clinical study of HD patients, a positive correlation was observed between serum sclerostin and phosphorus levels, and phosphorus was an independent factor affecting the level of sclerostin [32], which was also supported by our data. However, the regulatory relationship between calcium, phosphorus, vitamin D, and sclerostin remains to be determined.

This study had several limitations. First, this was a cross-sectional study with a relatively small sample size and we did not find clear evidence of a causal relationship between sclerostin and atherosclerosis. Second, the study involved only a single center in China, which limits the generalizability of the findings to other ethnic groups with different atherosclerosis risk profiles. Finally, we lacked histopathologic data from atherosclerotic plaques that could provide insight into the degree to which sclerostin affects plaque formation.

## Conclusion

In conclusion, the results presented here indicate that sclerostin level increases with the deterioration of renal function in patients with CKD 3–5ND, and that sclerostin plays a critical role in the development of atherosclerosis. Sclerostin is an independent risk factor for carotid atherosclerosis.

## Electronic supplementary material

Below is the link to the electronic supplementary material.

Supplementary file1 (XLS 74 kb)

## Abbreviations

ALP: Alkaline phosphatase
BMI: Body mass index
CKD: Chronic kidney disease
CKD-EPI: Chronic kidney disease-epidemiology collaboration equation
CKD-MBD: Chronic kidney disease–mineral bone disorder
CKD-ND: Non-dialysis patients with chronic kidney disease
CVD: Cardiovascular disease
DM: Diabetes mellitus
eGFR: Estimated glomerular filtration rate
ELISA: Enzyme-linked immunosorbent assay
FGF23: Fibroblast growth factor
HD: Hemodialysis
HDL-C: High-density lipoprotein cholesterol
Hs-CRP: High-sensitivity C-reactive protein
IDMS: Isotope dilution mass spectrometry
IMT: Intima-media thicknesses
iPTH: Intact parathyroid hormone
LDL-C: Low-density lipoprotein cholesterol
LRP6: Low-density lipoprotein receptor-related proteins 6
OR: Odds ratio
SD: Standard deviation
SOST KO: Sclerostin gene knockout
VSMCs: Vascular smooth muscle cells
WT: Wild-type
